# Glycerol Inhibition of Lactose‐Supported Growth and Lactose‐Driven Transcriptional Reprogramming in *Streptococcus pyogenes*

**DOI:** 10.1155/ijm/7188836

**Published:** 2026-11-09

**Authors:** Ayako Bando, Yujiro Hirose, Olivia M. Bertolla, Norihiko Takemoto, Stephan Brouwer, Jack O′Donohue, Masayuki Ono, Eri Ikeda, Victor Nizet, Mark J. Walker, Shigetada Kawabata

**Affiliations:** ^1^ Department of Microbiology Graduate School of Dentistry The University of Osaka Suita Osaka Japan osaka-u.ac.jp; ^2^ Australian Infectious Diseases Research Centre School of Chemistry and Molecular Biosciences and Institute for Molecular Bioscience The University of Queensland Brisbane Queensland Australia uq.edu.au; ^3^ Department of Infectious Diseases National Institute of Global Health and Medicine Japan Institute for Health Security (JIHS) Tokyo Japan; ^4^ Department of Pediatrics University of California at San Diego San Diego California USA ucsd.edu; ^5^ Skaggs School of Pharmaceutical Sciences University of California at San Diego San Diego California USA ucsd.edu

## Abstract

*Streptococcus pyogenes* (Group A *Streptococcus* [GAS]) requires flexible metabolic regulation to adapt to diverse host environments; however, its physiological responses to lactose and glycerol remain poorly characterized compared with glucose. In this study, we analyzed three representative serotype M1 GAS strains—the M1_global_ strain 5448, the ancestral M1 strain SF370, and SP1380 as a representative of the recently emerged M1_UK_ lineage. Glycerol consistently suppressed lactose‐dependent growth across all strains, whereas glycerol‐dependent respiratory activity was observed in 5448 and SF370 but was absent in SP1380. RNA‐seq analysis of strain 5448 revealed no significant transcriptional changes upon glycerol supplementation, indicating that this inhibitory effect likely occurs through a nontranscriptional mechanism. In contrast, lactose supplementation induced distinct transcriptional programs compared with glucose, including coordinated expression changes gene sets regulated by carbohydrate‐responsive transcription factors (CcpA, MalR, and NanR) and by the virulence regulator Mga and the stress‐response regulator Rgg. Together, these findings identify a previously unrecognized layer of carbon source‐dependent metabolic regulation and growth control in GAS.

## 1. Introduction

*Streptococcus pyogenes* (Group A *Streptococcus* [GAS]) is a *β*‐hemolytic gram‐positive bacterium that commonly colonizes the human nasopharynx and skin, particularly in children [1–3]. Although often asymptomatic, GAS is a major human pathogen responsible for diseases ranging from mild pharyngitis and impetigo to life‐threatening conditions such as necrotizing fasciitis and streptococcal toxic shock syndrome [4]. The M1 serotype is a predominant cause of invasive infections globally and in high‐income countries, making it essential to understand how this lineage adapts to various host niches to inform new approaches for effective control.

As a bacterium capable of both commensalism and invasive disease, GAS must deploy flexible metabolic strategies to survive in nutrient‐diverse environments. Carbohydrate utilization is central to GAS colonization and proliferation [5], and ~15% of its core genome encodes proteins involved in carbon metabolism [6]. GAS preferentially metabolizes glucose through glycolysis [7, 8]; however, glucose concentrations in the skin [9, 10] and nasopharynx [11]—its initial colonization sites—are markedly lower than in blood, resulting in limited glucose availability for bacterial metabolism. In the human pharynx [1, 2], GAS also intermittently encounters lactose derived from breast milk or dairy products, and glycerol is detectable in the oral cavity at low millimolar levels and is considered abundant [12]. Despite this, the growth behavior and transcriptional responses of GAS to lactose and glycerol remain largely uncharacterized.

Recent epidemiological studies have reported the global emergence and dissemination of a novel GAS M1 lineage, designated M1_UK_ [13–16]. This lineage has rapidly expanded in multiple countries, including the United Kingdom, the United States, and Australia, and is associated with increased incidence of scarlet fever and invasive streptococcal infections [17–19]. The M1_UK_ GAS lineage has been suggested to exhibit altered metabolic characteristics compared with the classical M1_global_ lineage. Genomic analyses have identified 27 lineage‐defining single‐nucleotide polymorphisms (SNPs) in M1_UK_, among which a defining feature is the increased expression of the streptococcal pyrogenic exotoxin A (SpeA) [20]. Notably, several SNPs occur in genes potentially involved in metabolism, including a nonsense mutation in *gldA* that introduces a premature stop codon and is predicted to disrupt glycerol dehydrogenase function [20]. In addition, a SNP within the promoter region of *glpF.2* (also known as *glA*), predicted to encode an aquaporin that may function as a glycerol transporter, has been shown to reduce the expression of this gene [15]. Together, these findings strongly suggest that glycerol metabolism has been remodeled in the M1_UK_ lineage. However, the physiological consequences of these metabolic differences, and how they impact the pathogenicity and adaptability of M1_UK_, remain poorly understood.

Based on these considerations, the present study is aimed at elucidating how lactose and glycerol influence the growth and metabolic regulation of GAS M1 serotype strains.

## 2. Materials and Methods

### 2.1. Strains

GAS serotype M1 strain 5448 is widely recognized as part of the modern globally disseminated M1 lineage, commonly termed the M1_global_ or M1T1 clone (Accession No. CP008776) [21]. By contrast, GAS M1 strain SF370 is regarded as an ancestral form of the M1 serotype. In the literature, SF370 is often described as an “archaic” or pre‐epidemic M1 lineage representative [22]. GAS serotype M1 strain SP1380 is a representative of the recently emerged M1_UK_ lineage, which is genetically distinct from the classical M1_global_ clone (Accession No. NZ_CP060269) [15]. Bacterial colonies grown on Tryptic Soy Agar supplemented with 5% sheep blood were inoculated into Todd–Hewitt broth supplemented with 2% yeast extract (THY; Hardy Diagnostics, Santa Maria, California, United States) and cultured overnight at 37°C in screw‐cap glass tubes under static conditions. The overnight cultures were used for subsequent experiments.

### 2.2. Growth Assay

Growth experiments were performed in a modified chemically defined medium (CDM), based on the formulation reported as previously described [23]. CDM was supplemented with glucose (4.5 g/L, 25.0 mM; G7021), lactose (4.5 g/L, 12.5 mM; 61339), or glycerol (4.5 g/L, 48.9 mM; G9012) as sole or combined carbon sources. All compounds were purchased from Sigma‐Aldrich (St. Louis, Missouri, United States). GAS cultures grown overnight in THY were diluted 1:5 into fresh THY and incubated at 37°C until they reached the mid‐exponential phase (OD_600_ = 0.4). Cells were harvested by centrifugation and resuspended in CDM without any carbon source. The resulting suspension was then diluted 1:5 into CDM containing the designated carbon sources. Growth kinetics were assessed in 96‐well microplates using a Tecan Infinite Pro 200 plate reader (Tecan, Männedorf, Switzerland). Plates were sealed and incubated at 37°C, with OD₆₀₀ measured at 30‐minute intervals.

### 2.3. Respiration Assay

To evaluate bacterial respiratory activity, we used Biolog Redox Dye H (Biolog Inc., Hayward, California, United States), which detects cellular respiration through colorimetric reduction. GAS cultures were grown overnight at 37°C in THY, and the overnight cultures were harvested by centrifugation and resuspended in CDM without any carbon source. The resulting suspension was then diluted 1:10 into CDM mixed with Redox Dye H at a final concentration of 1% (*v*/*v*). CDM was supplemented with glycerol (4.5 g/L, 48.9 mM). Respiration kinetics were monitored using a Tecan Infinite Pro 200 plate reader (Tecan, Männedorf, Switzerland) at 37°C, with OD_600_ measurements taken every 30 minutes. Microplates were sealed during incubation to prevent evaporation and maintain sterility.

### 2.4. RNA Extraction and Library Preparation

Overnight cultures in THY medium derived from different colonies were diluted 1:5 into fresh THY medium and incubated at 37°C, and growth was monitored by measuring the optical density at 600 nm. Mid‐exponential phase cells (OD_600_ = 0.4) were harvested by centrifugation, resuspended in CDM supplemented with the indicated carbon sources. For RNA extraction at 2 h, cultures were directly transferred to each carbohydrate condition at 1× concentration to obtain sufficient biomass. For RNA extraction at 5 h, cultures were inoculated at a 1:5 dilution and incubated under the same conditions. Although the inoculum size and growth state differed between experiments, these experiments were designed as independent comparisons rather than as direct comparisons between the 2 h and 5 h time points.

Each sample was then mixed with twice its volume of RNAprotect Bacteria Reagent (Qiagen, Hilden, Germany), vortexed, and incubated at room temperature for 5 min. After centrifugation, the supernatant was discarded, and total RNA was extracted from the resulting cell pellets using the Quick RNA Fungal/Bacterial Microprep kit (Zymo Research, Irvine, California, United States). Cells were disrupted mechanically for 30 s using a Multi Beads Shocker MB3000 (Yasui Kikai, Osaka, Japan). Library preparation was performed with the Ribo‐Zero Plus Microbiome kit and the TruSeq Stranded mRNA Library Prep kit (Illumina, San Diego, California, United States) according to the manufacturers′ protocols. Sample details of the 24 RNA samples and DEG information are provided in Data S1. All samples were collected in biological triplicates.

### 2.5. RNA‐Seq and Data Analysis

RNA sequencing and primary data analysis were performed at the NGS core facility of the Research Institute for Microbial Diseases, Osaka University. Libraries were prepared using the RiboZero Plus Microbiome kit followed by TruSeq Stranded mRNA workflow (Illumina) and sequenced on the NovaSeq X Plus platform (Illumina) using 151 bp paired‐end reads. Data processing and quality filtering were performed based on a previously published pipeline [24], with slight adaptations. Raw sequence reads were quality‐trimmed using fastp, and then mapped to the GAS strain 5448 genome using Bowtie2 [25]. Read counts were assigned to open reading frames (ORFs) using featureCounts [26]. Genes with an average count below 10 across samples were excluded to reduce noise from low‐expression features. Transcript abundance was normalized and expressed in transcripts per million (TPM). Differential gene expression and global transcriptomic analyses were conducted using iDEP [27]. For dimensionality reduction and clustering, EdgeR log transformation was applied, and hierarchical clustering was performed using the average linkage method with correlation distance.

RNA‐seq data generated in this study have been deposited in the DDBJ Sequence Read Archive (DRA) under BioProject accession number PRJDB37997, with individual sequencing runs assigned accession numbers DRR793594‐DRR793617. These data are currently under embargo and will be made publicly available in accordance with the DDBJ data release policy.

### 2.6. Data for iModulon Analysis and Code Availability

We utilized statistically independent components (M) previously identified through independent component analysis (ICA), along with normalized log TPM values (X) and their associated activity profiles (A) across various experimental conditions, all of which were obtained from iModulonDB [3, 8].

In this study, we further incorporated newly obtained normalized log TPM values (X) and the corresponding activity profiles (A), which were calculated using count data derived from bacterial RNA samples collected after 2 h of incubation. Details of these combined datasets are provided in Data S2. The code for assessment of the regulator enrichment can be found on Github (https://github.com/avsastry/modulome-workflow). Python package for analyzing and visualizing iModulons (PyModulon) can be also found on Github, (https://github.com/SBRG/pymodulon).

### 2.7. Quantification and Statistical Analysis

Statistical analysis was performed using GraphPad Prism Version 10.4.1. (GraphPad Software Inc., La Jolla, California, United States). Differences between groups were analyzed using a Mann–Whitney *U* test. Sample sizes and *p* values are indicated in figure legends.

### 2.8. Declaration of Generative AI and AI‐Assisted Technologies in the Writing Process

During the preparation of this work, the author(s) used ChatGPT 5.1 in order to assist with English‐language editing. After using this tool/service, the author(s) reviewed and edited the content as needed, and take full responsibility for the content of the publication.

## 3. Results

### 3.1. Glycerol Impairs Lactose‐Dependent Growth in All Three M1 Strains

To investigate how glycerol affects GAS growth on different carbohydrates, the M1_global_ type strain 5448, the ancestral strain SF370, and the newly emergent M1_UK_ clinical isolate SP1380 were cultured in CDM [23] supplemented with glucose, lactose, or glycerol, either individually or in combination (Figure 1A). In CDM lacking a carbon source (control) or supplemented with glycerol alone, no increase in optical density (OD_600_) was observed, indicating that glycerol cannot support growth under these conditions (Figure 1B–D). Although glycerol had no measurable effect on glucose‐dependent growth, it significantly suppressed lactose‐dependent growth—a phenotype consistently observed across all three strains (Figure 1B–D).

**Figure 1:**
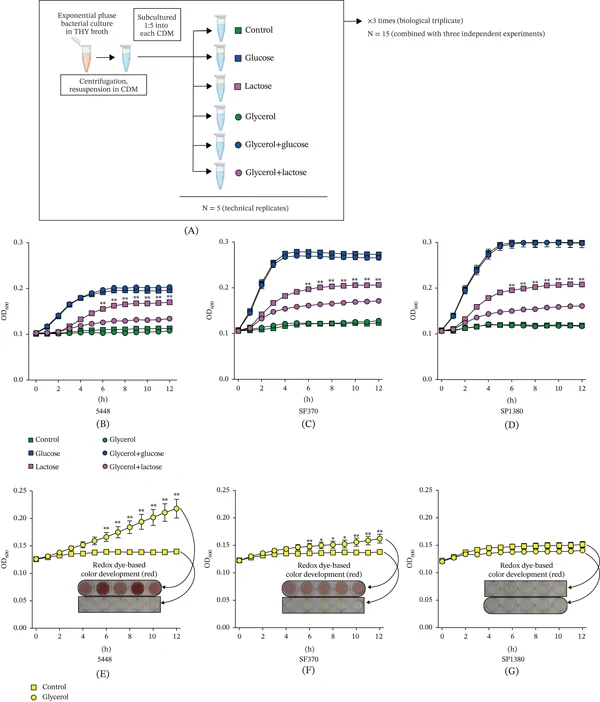
Growth of GAS in different carbohydrate conditions and glycerol‐induced respiratory activity. (A) Experimental workflow of the growth assay. Exponentially growing cultures in THY broth (Todd–Hewitt broth supplemented with 2% yeast extract) were centrifuged, resuspended in CDM, and subcultured into CDM supplemented with glucose, lactose, or glycerol, either individually or in combination. Growth curves were monitored under each condition. (B–D) Growth curves of the (B) M1 strain 5448, (C) the ancestral strain SF370, and (D) the M1_UK_ strain SP1380. Glycerol alone did not support growth, whereas glucose supported robust growth. Glycerol had no measurable effect on glucose‐dependent growth but significantly impaired lactose‐dependent growth in all three strains. (E–G) Respiratory activity assays for each strain. Cultures were incubated in CDM supplemented with Redox Dye H with or without glycerol, and OD_600_ was monitored using a plate reader. Glycerol induced a marked increase in respiratory activity in (E) strains 5448 and (F) SF370, but not in (G) SP1380. Representative images of redox dye‐based color development are shown below each graph. Experiments were performed in three independent biological replicates, each with five technical replicates (total *n* = 15). Error bars indicate the mean ± SEM. Statistical comparisons were performed for time points after 6 h. Statistical differences between groups were analyzed using the Mann–Whitney′s *U*‐test.  ^∗∗^ *p* < 0.01,  ^∗^ *p* < 0.05.

### 3.2. Glycerol Induces Respiration in 5448 and SF370 but Not in SP1380

Previous work from our group has demonstrated that strain 5448 can utilize glycerol to drive redox activity, as evidenced by the reduction of the redox‐sensitive dye Redox Dye H [23]. Although a different instrument and plate format were used in the present study, strain 5448 showed similar behavior (Figure 1E), exhibiting a time‐dependent increase in red color intensity, indicative of enhanced respiratory activity. Strain SF370 also actively respired glycerol in CDM supplemented with Redox Dye H (Figure 1F). In contrast, SP1380 displayed no change in color intensity under the same conditions, suggesting that glycerol does not trigger respiratory activity in this M1_UK_ lineage strain (Figure 1G).

These findings suggest that, compared with 5448 and SF370, strain SP1380 may have a defect in glycerol uptake or subsequent metabolic processing, consistent with prior genomic reports identifying a nonsense mutation in *gldA* and a promoter SNP in *glpF.2* (*glA*) that are predicted to impair glycerol metabolism [15, 20].

### 3.3. Glycerol Utilization Pathways in M1 Strains of GAS and Their Regulatory Disruption in Strain SP1380

According to NCBI sequence annotations, the major enzymes predicted to contribute to glycerol metabolism in GAS M1 strains are glycerol dehydrogenase (*gldA*), glycerol kinase (*glpK*), *α*‐glycerophosphate oxidase (*glpO*), and the glycerol transporters *glpF* and *glpF.2* (Figure 2A). To predict how these strains metabolize glycerol, we used our previously reported genome‐scale metabolic model of M1 GAS [23] together with the Escher visualization platform [28] to map metabolic routes from glucose, lactose, and glycerol to central metabolism (Figure 2B). The M1 GAS lineage (including strains SF370, 5448, and SP1380) encodes two aquaporin‐type glycerol transporters, GlpF and GlpF.2, the latter hypothesized to contribute to glycerol uptake. In the M1_UK_ lineage, a promoter SNP that reduces *glpF.2* (*glA*) expression and a nonsense mutation in *gldA.* Both mutations are present in SP1380 [15, 20].

**Figure 2:**
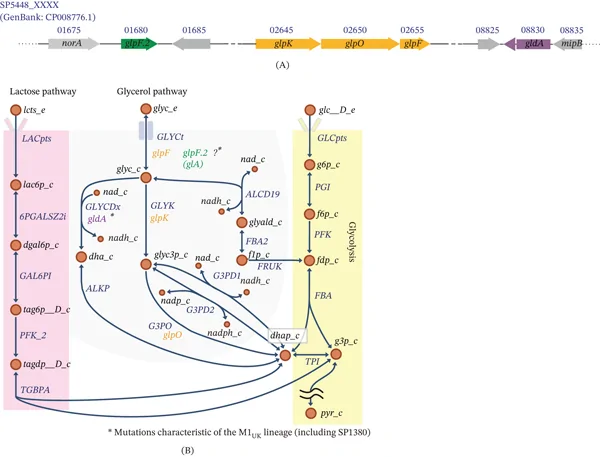
Genetic organization and metabolic overview of carbohydrate utilization in GAS serotype M1. (A) Genomic locus of genes involved in glycerol metabolism. (B) Overview of lactose, glucose, and glycerol metabolism in GAS serotype M1. Metabolite names are annotated with the suffix “_e” for extracellular and “_c” for cytoplasmic localization. Lactose pathway (pink background): Lactose (lcts_e) is transported into the cell via LACpts (PEP:pyruvate phosphotransferase system), forming lactose 6‐phosphate (lac6p_c). This intermediate is hydrolyzed by 6PGALSZ2i (6‐phospho‐*β*‐galactosidase) to produce 6‐phospho‐D‐galactose (dgal6p_c), which is subsequently isomerized by GAL6PI (galactose‐6‐phosphate isomerase) to yield D‐tagatose 6‐phosphate (tag6p__D_c). PFK_2 (tagatose‐6‐phosphate kinase) phosphorylates this compound to generate D‐tagatose 1,6‐bisphosphate (tagdp__D_c), which is then cleaved by TGBPA (tagatose‐bisphosphate aldolase) into glyceraldehyde 3‐phosphate (g3p_c) and DHAP (dhap_c), feeding into central metabolism. Glycerol pathway (gray background): Glycerol (glyc_e) is taken up into the cytoplasm via GLYCt encoded by *glpF*, resulting in cytoplasmic glycerol (glyc_c). In addition, *glpF.2* (*glA*) (aquaporin) has been suggested to contribute to glycerol transport, although this remains hypothetical. Glycerol can be metabolized through multiple oxidative routes: (i) GLYCDx (glycerol dehydrogenase, encoded by *gldA*) oxidizes glycerol to dihydroxyacetone (dha_c), which can then be converted to dhap_c by ALKP (alkaline phosphatase) as predicted in our model. (ii) GLYK (glycerol kinase, encoded by *glpK*) first phosphorylates glycerol to produce glycerol 3‐phosphate (glyc3p_c), which is then oxidized to dhap_c by G3PD1 or G3PD2 (glycerol‐3‐phosphate dehydrogenases). (iii) GLYK also phosphorylates glycerol to glyc3p_c, which can alternatively be oxidized by G3PO (glycerol 3‐phosphate oxidase, encoded by *glpO*) to generate dhap_c. (iv) ALCD19 (alcohol dehydrogenase) oxidizes glycerol to glyceraldehyde (glyald_c), which is further processed by FBA2 and FRUK to produce fructose 1,6‐bisphosphate (fdp_c) via fructose 1‐phosphate (f1p_c). Genes related to glycerol metabolism are labeled in green, orange, and purple: *glpF.2* (*glA*) in green, *glpK*, *glpO*, and *glpF* in orange, and *gldA* in purple. Asterisks indicate mutations characteristic of the M1_UK_ lineage, including SP1380: a nonsense mutation in *gldA* and reduced expression of *glpF.2* due to a promoter SNP have been reported [15]. Glucose pathway (yellow background): D‐glucose (glc__D_e) is taken up via GLCpts (glucose transport via PEP:Pyr PTS), phosphorylated to D‐glucose 6‐phosphate (g6p_c) by PGI (glucose‐6‐phosphate isomerase), then to D‐fructose 1,6‐bisphosphate (fdp_c) by PFK (phosphofructokinase). This is cleaved by FBA (fructose‐bisphosphate aldolase) into g3p_c and dhap_c. TPI (triose‐phosphate isomerase) interconverts g3p_c and dhap_c, which are then funneled into downstream energy metabolism, ultimately leading to the formation of pyruvate (pyr_c). Arrows indicate the direction of enzymatic reactions and metabolic flux.

Based on information from the genome‐scale model, glycerol can enter central metabolism through three potential routes In the first route, glycerol is sequentially processed by ALCD19 (alcohol dehydrogenase), FBA2 (D‐fructose‐1‐phosphate D‐glyceraldehyde‐3‐phosphate lyase), and FRUK (Fructose‐1‐phosphate kinase) to generate D‐fructose 1,6‐bisphosphate (fdp_c), a key glycolytic intermediate. In the second route, glycerol is phosphorylated by GLYK (glycerol kinase, *glpK*) to produce glycerol 3‐phosphate (glyc3p_c), which is then oxidized to dihydroxyacetone phosphate (DHAP) (dhap_c) by either G3PO (glycerol 3‐phosphate oxidase, *glpO*) or G3PD1/2 (glycerol‐3‐phosphate dehydrogenases, *gpsA*). In the third pathway, glycerol is oxidized directly to dihydroxyacetone (dha_c) by GLYCDx (glycerol dehydrogenase, *gldA*) and then converted to dhap_c. Because the M1_UK_ lineage strains carry a nonsense mutation in *gldA*, this branch is predicted to be impaired [20].

Collectively, these findings suggest that although M1 GAS strains possess multiple glycerol utilization pathways, their function may be disrupted in the M1_UK_ lineage. In SP1380, the absence of glycerol‐induced respiration (Figure 1G), together with loss of *gldA* function indicates that all three predicted glycerol metabolic routes may be nonfunctional.

### 3.4. Glycerol Does Not Influence the GAS Transcriptional Program

To evaluate transcriptional changes associated with glycerol uptake, we selected strain 5448, which exhibited the most pronounced glycerol‐dependent respiratory activity, for RNA‐seq analyses. To capture the early transcriptional responses to different carbon sources, RNA was extracted 2 h after inoculation (Figure 3A,B), a time point chosen to sensitively reflect carbon source‐dependent metabolic adaptation. In addition, because glycerol‐dependent inhibition of lactose‐supported growth became apparent later in culture, RNA was also collected at 5 h after inoculation for transcriptomic profiling (Figure 3D,E).

**Figure 3:**
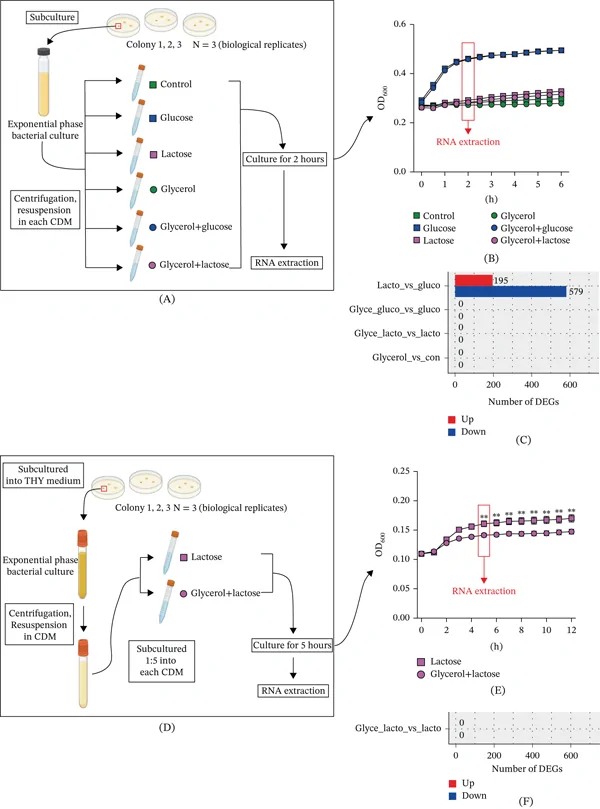
Transcriptomic profiling of GAS strain 5448 under defined carbon source conditions at 2 and 5 h postincubation. (A,B) Experimental design for transcriptomic analysis at 2 h. (A) Workflow of RNA‐seq experiments. Exponentially growing cultures of GAS 5448 were subcultured into CDM supplemented with glucose, lactose, glycerol, or their combinations (glycerol + glucose, glycerol + lactose). After 2 h of cultivation, cells were harvested for RNA extraction. Three independent biological replicates were performed. (B) Growth curve of the 5448 strain under the indicated conditions. The red arrow indicates the sampling point (2 h). At this time, cultures grown in glucose were in the late‐exponential phase, whereas cultures grown in other carbon sources (lactose, glycerol, or glycerol + lactose) remained in the lag phase. RNA was extracted at this point for transcriptomic analysis. Error bars indicate the mean ± SEM (*n* = 15). (C) Number of differentially expressed genes (DEGs) identified in each pairwise comparison (adjusted *p* < 0.1, 6). (D,E) Experimental design for transcriptomic analysis at 5 h. (D) Workflow of RNA‐seq experiments. Exponentially growing cultures of GAS 5448 were centrifuged, the supernatant was removed, and cells were resuspended in CDM. Cultures were then subcultured 1:5 into CDM supplemented with lactose or glycerol + lactose. After 5 h of cultivation, cells were harvested for RNA extraction. Three independent biological replicates were performed. (E) Growth curve of the 5448 strain under the indicated conditions. The red arrow indicates the sampling point (5 h). At this time, lactose‐dependent growth was suppressed by glycerol, and cells were harvested for RNA extraction for transcriptomic analysis. Error bars indicate the mean ± SEM (*n* = 15). Statistical comparisons were performed for time points after 5 h. Statistical differences between groups were analyzed using the Mann–Whitney′s *U*‐test.  ^∗∗^ *p* < 0.01. (F) DEGs identified between lactose and glycerol + lactose conditions (adjusted *p* < 0.1, |log*F* *C*| > 1).

At 2 h, no differentially expressed genes (DEGs) were identified when comparing glucose alone with glycerol + glucose, or lactose alone with glycerol + lactose (Figure 3C). Likewise, at 5 h, no DEGs were detected between lactose‐supplemented and glycerol + lactose‐supplemented conditions (Figure 3F). These findings indicate that glycerol does not measurably alter the global transcriptional profile, even at the time point when its inhibitor effect on glycerol‐dependent growth becomes evident.

In contrast, numerous DEGs were detected at 2 h when comparing glucose‐ and lactose‐supplemented conditions, demonstrating the induction of distinct transcriptional programs under these carbon sources (Figure 3C). Principal component analysis (PCA) and hierarchical clustering further revealed clear separation of samples grown in glucose versus lactose (Figure S1), confirming that global transcriptional profiles differ markedly between these conditions.

### 3.5. Transcriptional Changes and iModulon Activation in Lactose‐Supplemented Cultures Compared With Glucose

Substantial transcriptional differences were observed between glucose‐ and lactose‐supplemented cultures, with 195 genes significantly upregulated and 579 genes downregulated under lactose conditions (Figure 3C). A full list of significant DEGs is provided in Data S1. Exploring these differences may offer insights into why glycerol suppresses lactose‐dependent growth.

To investigate regulatory drivers of these transcriptional shifts, we examined iModulons*—sets of coexpressed genes identified through ICA and linked to specific regulators or* biological processes [8, 29–31]. In this framework, iModulon activity is calculated relative to the no‐carbon control (activity = 0), allowing direct comparison across conditions. Using Differential iModulon Activity (DiMA) analysis, we compared iModulon activation under glucose‐ versus lactose‐supplemented conditions (Figure 4A). Multiple iModulons associated with LacI family transcription factors—including CcpA, MalR, and NanR—showed increased activity under lactose conditions, consistent with the known roles of these regulators in responding to alternative sugars in the *Streptococcus* genus [32–34]. Additionally, increased activity was observed in iModulons associated with the virulence regulator Mga (involved in host adhesion and immune evasion) [35] and Rgg (involved in stress response) [36]. These results suggest that lactose availability triggers a broader coordinated regulatory response.

**Figure 4:**
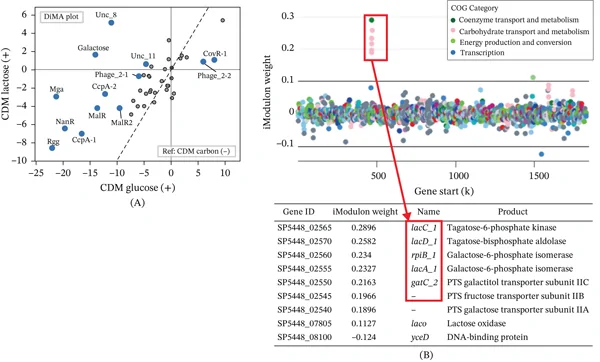
Differential iModulon activity (DiMA) analysis of GAS strain 5448 comparing lactose‐supplemented and glucose‐supplemented conditions, and genes comprising the Galactose iModulon. (A) The DiMA plot shows the comparison of iModulon activities between lactose‐ and glucose‐grown cultures, based on RNA‐seq data from three biological replicates per condition. iModulons associated with carbohydrate‐responsive transcription factors (CcpA, MalR, NanR) and host‐response regulators (Mga, Rgg) are activated. Notably, the Galactose iModulon also exhibits significant activation under lactose conditions. (B) Distribution and list of genes within the Galactose iModulon, including those involved in galactose and tagatose metabolism [3].

Galactose iModulon activity was also elevated under lactose conditions. Since lactose is metabolized intracellularly to lactose‐6‐phosphate, then to galactose‐6‐phosphate (Gal6P) before entering the tagatose‐6‐phosphate pathway (Figure 2B), induction of galactose‐associated metabolic genes is expected and consistent with the observed increase in Galactose iModulon activity. Consistent with this, the galactose‐associated metabolic genes *lacA*, *lacC*, and *lacD* were included in the Galactose iModulon (Figure 4B) [3].

Together, these findings demonstrate that shifting the available carbon source from glucose to lactose elicits extensive reprogramming of carbohydrate metabolism and regulatory networks, allowing the bacterium to maintain metabolic homeostasis under altered nutrient conditions.

## 4. Discussion

In this study, we investigated how lactose and glycerol influence growth and metabolic regulation in GAS. Among the three representative M1 strains (5448, SF370, and SP1380), glycerol consistently suppressed lactose‐dependent growth. Glycerol‐dependent respiratory activity was detectable in 5448 and SF370 but absent in SP1380. RNA‐seq analysis of 5448 revealed no significant transcriptional changes following glycerol supplementation, suggesting that this inhibitory effect occurs through a nontranscriptional mechanism. Transcriptomic profiling further showed that, compared with glucose‐supplemented conditions, lactose‐supplemented conditions induced distinct expression changes in multiple coexpressed gene sets (iModulons). These included modules associated with carbohydrate‐responsive regulators such as CcpA, MalR, and NanR [32–34], as well as iModulons linked to the virulence regulator Mga [35] and the stress‐response regulator Rgg [36]. Collectively, these findings suggest that, in the absence of glucose, lactose availability induces a coordinated transcriptional program that may enhance host adaptation and influence pathogenic potential.

In the M1_UK_ lineage, including SP1380 strain, a nonsense mutation in *gldA* and reduced expression of *glpF.2* (*glA*) have been reported [15, 20]. Given the absence of glycerol‐dependent respiration in SP1380 under our experimental conditions, it is possible that only the *gldA*‐mediated pathway remains functional among the three predicted routes for glycerol metabolism (Figure 2B). However, confirming this hypothesis will require additional studies using *gldA* deletion mutants in 5448 and SF370 backgrounds, which would allow direct comparison of glycerol utilization across the M1_global_, M1ancestral_,_ and M1_UK_ lineages. Taken together, these observations raise the possibility that glycerol utilization provided limited selective advantage in the ecological niche occupied by the M1_UK_ lineage, allowing the partial loss of glycerol metabolic functions during evolution. However, the evolutionary significance of this altered glycerol utilization phenotype remains unclear and warrants further investigation. Interestingly, glycerol suppressed lactose‐dependent growth in SP1380 despite the absence of detectable glycerol‐dependent respiratory activity. In addition, in strain 5448, glycerol suppressed lactose‐dependent growth without inducing transcriptional changes. One possible explanation for these observations is that extracellular glycerol interferes with the lactose transport system, although this possibility was not directly examined in the present study. Consistent with the potential importance of carbon source uptake for bacterial growth, the positive correlation between glucose uptake rate and growth rate has been reported in *Escherichia coli* [37].

As revealed by the DiMA analysis, activation of the Galactose iModulon was also observed under lactose‐supplemented conditions. According to iModulonDB (https://imodulondb.org/) [3], this iModulon includes *lacA*, *lacC*, and *lacD*, components of the *lacABCD* operon, which converts lactose‐derived intermediates—Gal6P and tagatose‐6‐phosphate—into glycolytic intermediates. This operon is strongly induced under lactose‐utilizing conditions and is conserved across many gram‐positive bacteria, including *Lactococcus lactis* and *Staphylococcus aureus*, where it contributes to lactose catabolism [38, 39]. In GAS, two distinct *lacABCD*‐containing loci exist, referred to as *lac.1* and *lac.2* operons, which have been reported to contribute to lactose‐dependent growth [40]. These observations are consistent with the metabolic map in Figure 2B and support the model in which GAS uses the galactose‐tagatose pathway to convert lactose into intermediates feeding into glycolysis. Interestingly, the *lac.1* genes were included in the CcpA‐2 and Galactose iModulons, whereas the *lac.2* genes were included in the Mga iModulon. All three iModulons were activated under lactose conditions, suggesting roles for CcpA and Mga in regulating lactose‐responsive genes. This is consistent with prior work showing that CcpA coordinates virulence and metabolic gene expression in response to carbohydrate availability in GAS [41]. Moreover, the concurrent activation of CcpA‐ and Mga‐associated iModulons suggests that CcpA may indirectly influence Mga activity through metabolic signaling, likely by altering intracellular carbohydrate flux or energy status. This supports the model in which CcpA integrates metabolic control with virulence gene regulation [41]. Together, these findings support a model in which CcpA‐ and Mga‐mediated regulatory networks cooperatively control transcriptional responses to lactose in GAS.

From a metabolic perspective, although lactose metabolism also provides some glucose‐derived carbon that enters glycolysis upstream, bacterial growth under lactose conditions does not reach the level achieved with glucose alone. Lactose, a disaccharide of glucose and galactose, is metabolized in GAS into glucose‐6‐phosphate (G6P) and Gal6P, respectively. G6P can be directly funneled into glycolysis, supporting efficient ATP production. In contrast, Gal6P enters the tagatose‐6‐phosphate pathway and is converted into DHAP and glyceraldehyde‐3‐phosphate (G3P), which merge into glycolysis at downstream steps. Considering this pathway, lactose metabolism should, in principle, be sufficient to support bacterial growth. However, our RNA‐seq analysis revealed that the expression of several genes encoding upper glycolytic enzymes—including *pgi* (G6P isomerase), *pfkA* (6‐phosphofructokinase), and *fba* (fructose‐bisphosphate aldolase Class II)—was decreased under lactose‐supplemented conditions compared with glucose‐supplemented conditions (Data S2). These enzymes are responsible for maintaining the levels of glycolytic intermediates such as G6P and fructose‐6‐phosphate (F6P), which are essential for both energy generation and cell wall biosynthesis. Therefore, impaired synthesis of these intermediates may underlie the reduced growth observed under lactose‐supplemented conditions. The underlying factors may not be fully explained by pathway architecture alone and will require further investigation.

Several limitations of this study should be acknowledged. First, the CDM used in this study was designed to facilitate the detection of carbon source‐dependent metabolic responses and was not intended to faithfully reproduce the nutritional environment of the human pharynx or skin. In the oral cavity, glycerol is present at low millimolar concentrations [12], whereas exposure to glucose and lactose through dietary intake is likely to be intermittent. In addition, GAS in vivo is likely exposed to multiple carbon sources rather than to the defined carbon‐source conditions used in this study. Therefore, the growth‐inhibitory effect of glycerol observed under these defined conditions may not fully reflect its impact in vivo. Second, although all experimental groups were subjected to the same pregrowth conditions, the potential influence of prior glucose exposure cannot be completely excluded, as glucose is known to regulate gene expression through carbon catabolite repression (CCR) in GAS [42]. In addition, the possible presence of trace amounts of glucose in the high‐purity lactose preparation cannot be completely excluded because its glucose content was not independently quantified. Finally, transcriptomic analysis was performed using only the representative M1 strain 5448. Although the growth phenotypes associated with lactose and glycerol utilization were observed across multiple GAS strains, the extent to which the transcriptional responses identified in strain 5448 are conserved among other genetic backgrounds remains to be determined.

## 5. Conclusion

In this study, we demonstrated that glycerol specifically suppresses lactose‐dependent growth in GAS through a mechanism that appears independent of transcriptional changes. Although we predicted carbon source‐specific metabolic pathways and examined the associated metabolic and regulatory responses under different sugar conditions, the precise mechanism by which glycerol inhibits lactose‐supported growth could not be identified. Nevertheless, the marked transcriptional reprogramming observed when the carbon source shifted from glucose to lactose indicates that GAS activates coordinated regulatory programs and flexibly modulates its metabolism according to carbohydrate availability. These findings reveal a previously unrecognized aspect of metabolic control in this pathogen and provide an important foundation for deeper investigation into the nutrient‐dependent infection strategies of GAS.

## Funding

This study was supported by the Ministry of Health, Labour and Welfare (10.13039/501100003478); Japan Agency for Medical Research and Development (10.13039/100009619; JP23wm0325066); Japan Society for the Promotion of Science (10.13039/501100001691; 26K23602, 25K00123, 25K02804, 23KK0281); JST SPRING (JPMJSP2138); Takeda Science Foundation (10.13039/100007449); and Chemo‐Sero‐Therapeutic Research Institute.

## Conflicts of Interest

The authors declare no conflicts of interest.

## Supporting information


**Supporting Information** Additional supporting information can be found online in the Supporting Information section. Figure S1 presents additional transcriptional analyses. Data S1 contains metadata and differential gene expression data. Data S2 contains transcriptomic datasets and iModulon analysis results. Figure S1: Principal component analysis and hierarchical clustering of transcriptomes of GAS strain 5448 under different carbon source conditions. (A) Principal component analysis (PCA) of global expression across the six conditions: control (no carbon), glucose, lactose, glycerol, glycerol + glucose, and glycerol + lactose. Samples supplemented with glucose (with or without glycerol) cluster closely, whereas other conditions show greater dispersion. (B) Hierarchical clustering of transcriptomic profiles from the same six conditions. Clustering was based on the top 1000 most variable genes. Data S1: This file contains two sheets: (1) metadata of 24 RNA‐seq samples, and (2) differentially expressed genes (DEGs) from comparisons between CDM glucose (+) and CDM lactose (+) conditions. Data S2: This file contains three sheets: (1) log TPM values (expression data, X) used in this study, (2) the M‐matrix representing gene weightings in each of the 42 iModulons (output of independent component analysis), and (3) the A‐matrix representing the activity of each of the 42 iModulons.

## Data Availability

The RNA‐seq data generated in this study have been deposited in the DDBJ Sequence Read Archive (DRA) under BioProject Accession Number PRJDB37997. These data are currently under embargo and will be made publicly available in accordance with the DDBJ data release policy.
