# Supplementary material for: Glycerol Inhibition of Lactose‐Supported Growth and Lactose‐Driven Transcriptional Reprogramming in Streptococcus pyogenes
Source: Int J Microbiol. 2026 Nov 9;2026:7188836. doi: 10.1155/ijm/7188836 (PMC13748936; doi:10.1155/ijm/7188836)
Supplement: Supplementary file 1 [file IJM-2026-7188836-s001.zip › 7188836.f1/GlyceLacto_SupplementaryFigure20261029.docx]

**Glycerol inhibition of lactose-supported growth and lactose-driven transcriptional reprogramming in *Streptococcus pyogenes***

**Authors**

Ayako Bando¹, Yujiro Hirose¹*, Olivia M. Bertolla², Norihiko Takemoto³, Stephan Brouwer², Jack O’Donohue², Masayuki Ono¹, Eri Ikeda¹, Victor Nizet⁴⁵, Mark J. Walker², Shigetada Kawabata¹

**Affiliations**

¹Department of Microbiology, Graduate School of Dentistry, The University of Osaka, Suita, Osaka

²Australian Infectious Diseases Research Centre, School of Chemistry and Molecular Biosciences, and Institute for Molecular Bioscience, The University of Queensland, Brisbane, QLD

³Department of Infectious Diseases, National Institute of Global Health and Medicine, Japan Institute for Health Security (JIHS), Tokyo

⁴Department of Pediatrics, University of California at San Diego, La Jolla, California

⁵Skaggs School of Pharmaceutical Sciences, University of California at San Diego, La Jolla, California

*Correspondence:

Yujiro Hirose, E-mail: hirose.yujiro.dent@osaka-u.ac.jp, TEL: +81668792897

**Supplementary Figure**

**Figure S1. Principal component analysis and hierarchical clustering of transcriptomes of *S.pyogenes* strain 5448 under different carbon source conditions.**

(A) Principal component analysis (PCA) of global expression across the six conditons:

Control (no carbon), glucose, lactose, glycerol, glycerol + glucose, and glycerol + lactose. Samples supplemented with glucose (with or without glycerol) closely, while other conditions show greater dispersion.

(B) Hierarchical clustering of transcriptomic profiles from the same six conditions. Clustering was based on the top 1,000 most variable genes.
